# Burn‐in‐Loss‐Free Organic Solar Cells With Over 2000 h of Thermal Stability Through Nitrone‐Based Interface Passivation

**DOI:** 10.1002/smll.74851

**Published:** 2026-07-31

**Authors:** Sanseong Lee, Kiyoung Park, Changhoon Lee, Oskar J. Sandberg, Ju‐Hyeon Kim, Taeyoon Ki, Jong‐Hoon Lee, Dongguen Shin, Jong Sung Jin, Ji Yeong Sung, In‐Wook Hwang, Jubin Jang, Kitae Kim, Soohyung Park, Ji Hoon Shim, Hongkyu Kang, Kwanghee Lee

**Affiliations:** ^1^ Heeger Center for Advanced Materials Gwangju Institute of Science and Technology Gwangju Republic of Korea; ^2^ Research Institute of Solar and Sustainable Energies (RISE) Gwangju Institute of Science and Technology Gwangju Republic of Korea; ^3^ College of Engineering Department of Materials Science and Engineering Gwangju Institute of Science and Technology Gwangju Republic of Korea; ^4^ Max Planck–POSTECH Center for Complex Phase Materials Pohang University of Science and Technology Pohang Republic of Korea; ^5^ Division of Advanced Materials Science Pohang University of Science and Technology Pohang Republic of Korea; ^6^ Physics, Faculty of Science and Engineering Åbo Akademi University Turku Finland; ^7^ Department of Physics, Chemistry and Biology (IFM) Linköping University Linköping Sweden; ^8^ Department of Advanced Materials Engineering Kyonggi University Suwon Republic of Korea; ^9^ Department of Physics Chonnam National University Gwangju Republic of Korea; ^10^ Busan Center Korea Basic Science Institute (KBSI) Busan Republic of Korea; ^11^ Advanced Photonics Research Institute Gwangju Institute of Science and Technology Gwangju Republic of Korea; ^12^ Advanced Analysis and Data Center Korea Institute of Science and Technology (KIST) Seoul Republic of Korea; ^13^ Department of Chemistry Pohang University of Science and Technology Pohang Republic of Korea

**Keywords:** molybdenum oxide, nitrone, organic solar cells, passivation, thermal stability

## Abstract

Despite the remarkable advancement of organic solar cells (OSCs) with power conversion efficiencies (PCEs) surpassing 20%, the thermal instability at interfacial layers remains a major obstacle to their practical commercialization. In particular, the molybdenum oxide (MoO_x_) hole‐transport layer in inverted OSCs (I‐OSCs) is prone to thermally induced degradation, including work function (WF) shallowing and interfacial diffusion. Although several passivation strategies have been explored to mitigate MoO_x_ instability, their effectiveness remains limited, and the development of advanced passivation materials is still lacking. Here, we report a nitrone‐based passivation strategy using N‐tert‐butyl‐α‐phenylnitrone (PBN). The polar nitrone (N^+^–O^−^) moiety facilitates strong interactions with under‐coordinated molybdenum sites, suppressing both WF shifts and diffusion of MoO_x_. Notably, comprehensive analyses, including OrbiSIMS depth profiling and drift‐diffusion modeling, reveal that MoO_x_ diffusion, rather than WF shallowing, is the dominant degradation pathway. Consequently, PBN‐passivated devices retain ∼100% of their initial PCE (∼18.1%) after 2000 h at 85°C without a rapid initial PCE drop, known as burn‐in loss. The general applicability of this strategy is further confirmed in PM6:L8BO, PM6:Y6, and PM6:BTP‐eC9 BHJ systems, offering a viable pathway for fabricating robust I‐OSCs.

## Graphical abstract

A nitrone‐based molecular passivation strategy enhances the thermal stability of MoO_x_‐based inverted organic solar cells. The polar N^+^–O^−^ group of N‐tert‐butyl‐α‐phenylnitrone passivates under‐coordinated molybdenum sites, suppressing oxygen vacancy formation and MoO_x_ diffusion into the bulk heterojunction. This interface engineering preserves charge extraction and maintains device efficiency across multiple donor–acceptor systems under prolonged thermal stress.

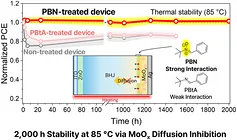

## 1 Introduction

Organic solar cells (OSCs) have attracted considerable attention as a promising photovoltaic technology, with power conversion efficiencies (PCE) now exceeding 20% owing to advances in photoactive material design and interfacial layer optimization [1, 2, 3]. However, their long‐term operational stability remains a critical bottleneck for commercialization. While extrinsic degradation factors, such as moisture, oxygen, and UV exposure, can be mitigated through encapsulation or optical filters [4, 5], the elevated operating temperature of OSCs (typically 60°C–65°C) [6] inevitably induces morphological instability and interfacial degradation [7, 8].

Remarkable progress has been achieved in mitigating thermal degradation through morphological engineering of the bulk heterojunction (BHJ) layer, specifically by incorporating high‐glass‐transition‐temperature (T_g_) photoactive materials or morphology stabilizers to prevent heat‐induced phase separation [9, 10, 11]. However, even with a morphologically stable BHJ layer, thermal degradation of the interlayer can significantly impede charge extraction to the corresponding electrodes, leading to severe performance deterioration [12, 13]. Therefore, investigating the thermal degradation mechanisms of interfacial layers and developing stabilization strategies are prerequisites to ensure the long‐term operation of OSCs.

Given the critical importance of interfacial thermal stability, inverted structure OSCs (I‐OSCs) are widely recognized as more thermally stable than conventional structure OSCs [14], primarily because they usually utilize inorganic metal oxides—such as ZnO and molybdenum oxide (MoO_x_)—as interlayers rather than the thermally unstable organic interlayers commonly employed in conventional OSCs (e.g., 2PACz, PFN‐Br, and BCP) [15, 16, 17]. However, evaporated MoO_x_, the standard hole‐transport layer in I‐OSCs, exhibits inherent thermal vulnerability, typically attributed to two primary degradation pathways: (i) thermal generation of oxygen vacancies, which reduces work function (WF) and thus impedes hole extraction [18, 19], and (ii) diffusion of MoO_x_ species into the BHJ layer, acting as p‐dopants and charge recombination traps [20, 21, 22]. Despite these known mechanisms, systematic investigations comparing the relative contributions of each pathway to device performance loss are still lacking.

To date, significant efforts have been made to suppress the degradation using vacuum‐deposited nitrogen‐containing aromatic organic materials (e.g., BCP, TPBi, and TCTA) as an interlayer between the MoO_x_ and photoactive layers [21, 23, 24]. These materials have proven effective in forming diffusion barriers, and it has been suggested that they may contribute to MoO_x_ surface modification through chemical interactions involving their nitrogen lone pairs. However, the effectiveness of such interactions might be limited in bulky aromatic systems due to resonance delocalization, which diminishes the electron‐donating capability of the nitrogen lone pairs, or steric hindrance that impedes a close approach to the active sites on the MoO_x_ surface [25]. Furthermore, this approach increases fabrication complexity by introducing an additional vacuum step. More importantly, achieving an effective barrier against MoO_x_ diffusion often necessitates thicker interlayers that decrease initial PCE; nevertheless, further improvements are still required to ensure sufficient thermal stability for long‐term operation [21, 24].

Herein, we report a facile, solution‐processable interface engineering strategy to enhance the thermal stability of MoO_x_‐based I‐OSCs using N‐tert‐butyl‐α‐phenylnitrone (PBN). The highly polar nitrone moiety (N^+^‐O^−^) of PBN functions as a strong Lewis base, enabling strong interfacial interactions with under‐coordinated molybdenum (Mo) sites, which serve as electropositive centers. This specific interaction effectively passivates surface defects and suppresses the formation of oxygen vacancies under thermal stress, thereby mitigating the degradation of MoO_x_‐based OSCs. The critical role of the nitrone functionality in PBN is further verified by comparing its passivation effect with that of a structural analogue, N‐tert‐butyl‐1‐phenylmethanimine (PBtA). Furthermore, systematic analysis reveals that MoO_x_ diffusion, rather than WF shallowing, is the dominant driver of thermal failure. Consequently, our PBN‐passivated devices exhibit remarkable thermal stability, maintaining nearly 100% of their initial efficiency after 2000 h at 85°C, without compromising initial PCE relative to the control devices. Finally, we extended the applicability of this strategy from the representative PM6:L8BO system to other high‐performance non‐fullerene acceptor (NFA) systems (PM6:Y6 and PM6:BTP‐eC9), thereby demonstrating its potential as a general approach for achieving long‐term operational stability in OSCs.

## 2 Results and Discussion

### 2.1 Thermal Degradation Mechanism and Passivation Strategy

**SCHEME 1:**
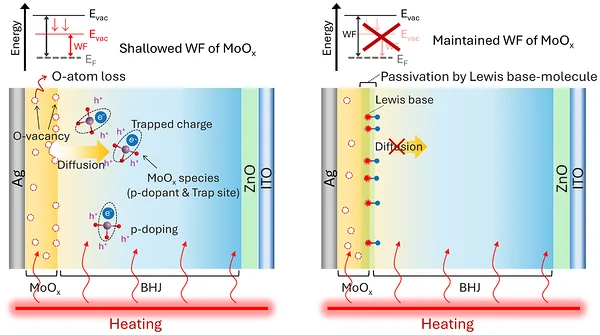
Mechanism for the thermal degradation of MoO_x_‐based I‐OSCs and the stabilizing effect of a Lewis‐base passivation layer. Left: Under thermal stress, the MoO_x_/BHJ interface degrades through oxygen atom loss, resulting in a reduced MoO_x_ work function (WF) and the diffusion of MoO_x_ species into the BHJ. Right: The introduction of a Lewis‐base molecular layer on the MoO_x_ surface effectively passivates active sites, inhibiting oxygen vacancy formation and thereby stabilizing the MoO_x_ layer.

Scheme 1 illustrates the proposed thermal degradation mechanism in MoO_x_‐based I‐OSCs and the corresponding stabilization strategy using a Lewis‐based passivator. Under thermal stress, MoO_x_ undergoes oxygen loss, leading to the formation of oxygen vacancies and the reduction of Mo^6+^ to Mo^5+^/Mo^4+^ states. The oxygen vacancies generate electron‐filled gap states below the conduction band and lower the WF of MoO_x_, which weakens the interfacial band bending at the MoO_x_/BHJ interface and reduces the built‐in potential. Because MoO_x_ is an n‐type semiconductor, the diminished band bending decreases the electron blocking barrier, thereby impairing hole selectivity and facilitating electron leakage [26, 27, 28]. Concurrently, oxygen vacancy‐induced changes can undermine the structural stability of the MoO_x_ surface, potentially facilitating the generation of mobile MoO_x_ species that can diffuse into the BHJ layer and act as p‐dopants and deep trap sites, thereby degrading the device performance [22, 29, 30]. The right panel of Scheme 1 illustrates the mechanism of MoO_x_ interface stabilization, in which a strong Lewis base forms robust interactions with under‐coordinated Mo sites [31]. This passivation strategy can suppress oxygen vacancy‐induced WF reduction and MoO_x_ species diffusion.

### 2.2 Electronic and Chemical Characterization of the Passivator‐MoO_x_ Interface

**FIGURE 1:**
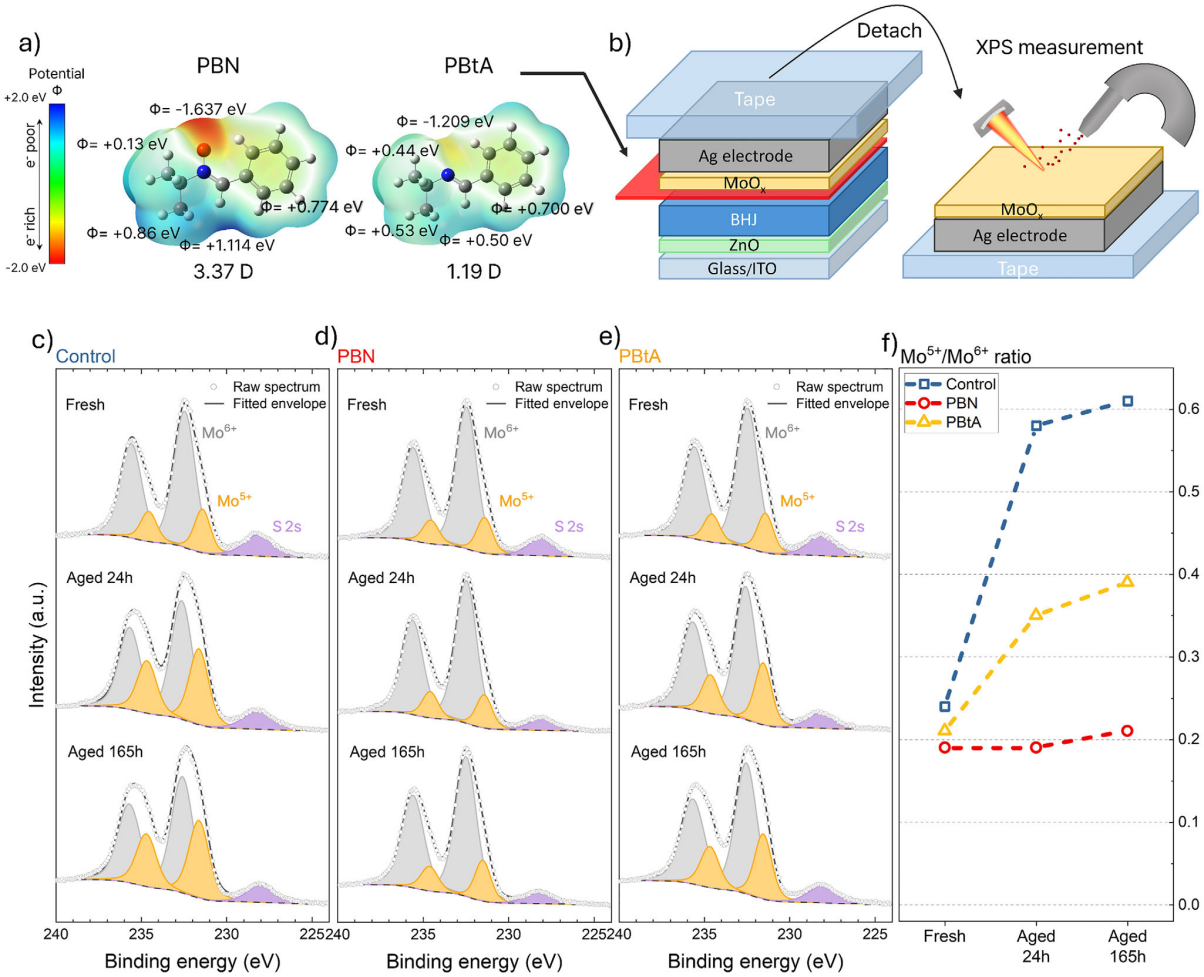
(a) Calculated electrostatic potential maps for PBN and PBtA molecules. The constituent atoms are color‐coded as follows: grey, carbon; white, hydrogen; blue, nitrogen; and red, oxygen. (b) Schematic of the mechanical delamination process for probing the buried MoO_x_ interface via XPS. (c–e) Evolution of Mo 3d XPS spectra for (c) Control, (d) PBN, and (e) PBtA samples under thermal stress (85°C). The raw spectra (open circles) were deconvoluted into Mo^6^^+^ (gray), Mo^5^^+^ (yellow), and S 2s (purple, from BHJ residue on the delaminated surface) components. (f) Corresponding evolution of the Mo^5^^+^/Mo^6^^+^ peak area ratio calculated from the fitted spectra, indicating the degree of chemical reduction at the interface.

To implement the passivation mechanism proposed in Scheme 1, a potent Lewis base is required to interact strongly with the electropositive under‐coordinated Mo cations at oxygen vacancy sites. We hypothesize that the nitrone moiety in PBN can act as a strong Lewis base and facilitate such interfacial interactions. To verify this, the electronic properties of PBN, specifically the high polarity of its nitrone (N^+^–O^−^) functional group, were compared with those of its imine‐based analogue (PBtA), focusing on the charge distribution around the nitrone/imine moiety. As shown in Figure 1a, PBN exhibits a markedly more negative electrostatic potential at the nitrone oxygen (Φ ≈ −1.637 eV) and a larger dipole moment (µ = 3.37 D) relative to PBtA (Φ ≈ −1.209 eV, µ = 1.19 D). This theoretical analysis supports that the nitrone group in PBN likely induces stronger interactions with the under‐coordinated Mo sites on the MoO_x_ surface.

To assess the efficacy of PBN in stabilizing the MoO_x_ interface against thermal stress, x‐ray photoelectron spectroscopy (XPS) analysis was performed. The MoO_x_ layer was mechanically delaminated in a N_2_‐filled glovebox and immediately vacuum‐transferred to an XPS chamber to prevent air exposure (Figure 1b and Figure S1). Figure 1c–e presents the Mo 3d core‐level spectra of the Control (untreated), PBN, and PBtA samples, respectively, under thermal stress (fresh, 24 h, and 165 h at 85°C). Peak deconvolution revealed two primary oxidation states: Mo^6+^ (stoichiometric lattice, Mo 3d_5/2_ and 3d_3/2_ at 232.4 and 235.6 eV) and Mo^5^^+^ (oxygen‐deficient sites, Mo 3d_5/2_ and 3d_3/2_ at 231.4 and 234.5 eV) components.

For the Control sample (Figure 1c), the Mo 3d spectra showed progressive broadening with thermal aging, accompanied by an increase in the Mo^5+^ component. The Mo^5^^+^/Mo^6^^+^ ratio increased sharply from 0.24 (fresh) to 0.58 (24 h) and 0.61 (165 h), confirming thermal‐induced formation of oxygen vacancies (Figure 1f). Concurrently, the component peaks shifted to higher binding energies by ∼+0.09 eV (24 h) and +0.12 eV (165 h) on average, indicating a decrease in the WF of MoO_x_ [28, 32], which is a direct consequence of n‐doping by oxygen vacancies. In contrast, the PBN sample (Figure 1d) demonstrated outstanding chemical and electronic stability. The fresh PBN sample exhibited a lower initial Mo^5+^/Mo^6+^ ratio (∼0.19) compared with the Control, suggesting that PBN passivated the pre‐existing oxygen vacancies formed during MoO_3_ evaporation [33, 34]. More importantly, this stoichiometric integrity was preserved even after 165 h of thermal stress, with negligible changes in both the Mo^5+^/Mo^6+^ ratio (Figure 1f) and binding energy. Comparatively, PBtA (Figure 1e) was far less effective than PBN in suppressing oxygen vacancy generation.

To further examine the involvement of the nitrone moiety in the PBN–MoO_x_ interaction, Fourier‐transform infrared (FTIR) measurements were additionally performed using a MoO_x_/PBN structure, in which PBN was spin‐coated onto a pre‐deposited MoOx film (Figure S2). Upon contact with MoOx, the nitrone‐associated v(N–O) band reproducibly shifted from 1076 to 1078 cm^−1^, accompanied by a small shift of the adjacent v(C–N) band from 1118 to 1119 cm^−1^, whereas the reference vibrations assigned to v(C(CH_3_)_3_) at 1194 cm^−1^ and v(C–H) at 905 cm^−1^ remained essentially unchanged [35, 36]. These selective spectral changes suggest that the nitrone moiety is involved in the interfacial interaction with MoO_x_.

### 2.3 Computational Analysis of the PBN Stabilization Mechanism

**FIGURE 2:**
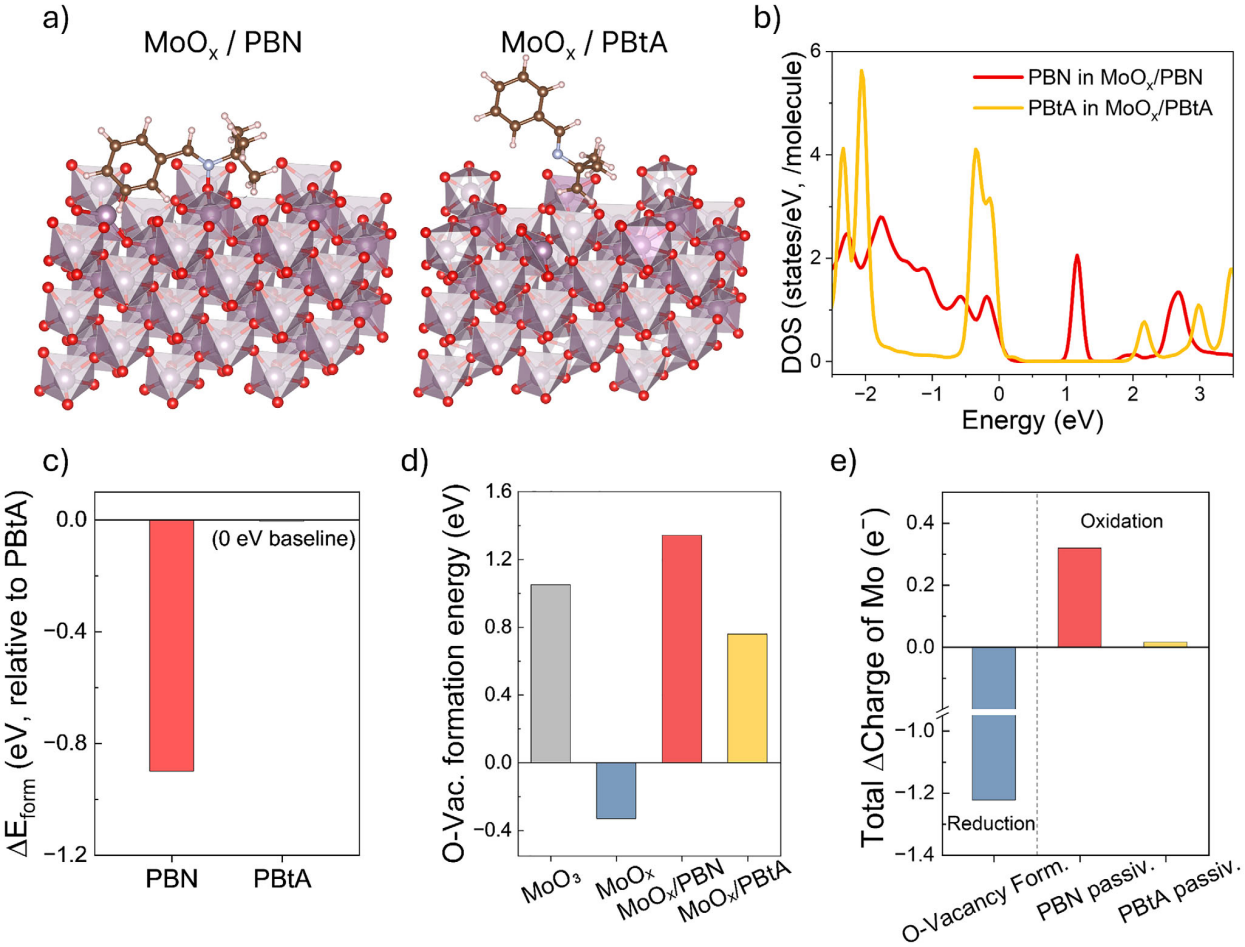
(a) Optimized adsorption geometries of PBN and PBtA on the defective MoO_x_ surface. (b) Projected density of states (DOS) of PBN and PBtA adsorbed on MoO_x_. (c) Calculated adsorption energy (ΔE_form_) of PBN on MoO_x_, shown relative to the PBtA baseline (0 eV). (d) Calculated oxygen vacancy formation energy for pristine MoO_3_, defective MoO_x_, PBN‐passivated MoO_x_ (MoO_x_/PBN), and PBtA‐passivated MoO_x_ (MoO_x_/PBtA). (e) Total Bader charge difference of the 15 surface Mo atoms upon oxygen vacancy formation and subsequent PBN or PBtA passivation.

Comprehensive density functional theory (DFT) calculations were performed to elucidate the atomistic and electronic origins of the remarkable stabilizing effect of PBN. The interaction of PBN or PBtA with a defective MoO_x_ surface was investigated by modeling oxygen vacancy sites, which showed significant thermal‐induced evolution in our XPS analysis. First, we investigated the adsorption geometries of PBN and PBtA on defective MoO_x_ surfaces (Figure 2a). Although the imine nitrogen in PBtA is electron‐rich (Figure 1a), it is sterically hindered by the bulky tert‐butyl and phenyl groups, making it difficult to interact directly with the vacancy site. The optimized geometry (Figure 2a, right) confirms this, showing that the tert‐butyl group is anchored to the surface, leading to a non‐ideal adsorption configuration. In contrast, the nitrone oxygen in PBN is sterically accessible (protruding from the molecular backbone), allowing favorable interaction with the under‐coordinated Mo cation at the vacancy site (Figure 2a, left).

To explore how molecular passivation modifies the defect‐induced electronic structure of MoO_x_, we calculated the Mo‐projected density of states (DOS) of the pristine MoO_3_, oxygen‐deficient MoO_x_, passivated MoO_x_/PBN, and MoO_x_/PBtA surfaces (Figure S3). Oxygen‐deficient MoO_x_ exhibits pronounced mid‐gap states within 0.5–1.7 eV above the Fermi level, whereas interaction with either PBN or PBtA substantially reduces these states, indicating that both molecules provide a certain degree of electronic passivation of oxygen vacancies. To rationalize why PBN yields markedly stronger stabilization, we further examined the projected DOS of the adsorbed molecules (Figure 2b). PBN showed broad, delocalized features over a wide energy range, indicating strong hybridization and efficient delocalization of vacancy‐related electrons, while PBtA retained sharp, localized peaks, indicating weaker coupling and less effective stabilization.

To determine the passivation preference and stability, we evaluated the relative formation energy, ΔE_form_ = E(MoO_x_ + molecules) – E(MoO_x_) – E(molecule) (Figure 2c). Using PBtA as the baseline (0 eV), PBN exhibits a relative formation energy of ∼–1.2 eV, indicating that its adsorption is substantially more exothermic and thermodynamically favorable than PBtA. This suggests that the enhanced interaction of PBN originates from the sterically accessible and chemically reactive nitrone functionality, which enables stronger interaction and pronounced electronic hybridization with the defective MoO_x_ surface.

To assess thermodynamic stabilization against defect formation, we calculated the oxygen vacancy formation energy (OVFE)—a key metric for degradation resistance—by removing oxygen atoms at six surface oxygen sites (positions shown in Figure S4). Pristine MoO_3_ exhibits a positive OVFE (+1.05 eV), indicating that oxygen loss is thermodynamically unfavorable (Figure 2d). However, in oxygen‐deficient MoO_x_, oxygen removal becomes thermodynamically favorable, as reflected by the negative OVFE (−0.33 eV), providing a thermodynamic driving force for additional vacancy formation. Remarkably, PBN passivation increases OVFE to +1.34 eV, demonstrating that PBN not only blocks existing vacancy sites but also chemically reinforces the MoO_x_ lattice, thermodynamically suppressing vacancy formation and propagation. In contrast, PBtA passivation offers only moderate stabilization, with an OVFE value of +0.76 eV, consistent with its limited efficacy in suppressing the oxygen vacancy generation, as observed in the XPS analysis.

A Bader charge analysis was performed to elucidate the underlying electronic mechanism (Figure 2e). The formation of an oxygen vacancy in MoO_x_ is a reductive process, wherein adjacent Mo atoms acquire ∼1.2 e^−^. Upon PBN passivation, these reduced Mo atoms are partially re‐oxidized, transferring 0.32 e^−^ to the PBN molecule. In other words, PBN behaves as an electron‐withdrawing ligand through its nitrone oxygen, interacting with exposed Mo (a Lewis acid at the vacancy site) and delocalizing excess electrons across its phenyl–nitrone core (Figure S5). This electronic interaction is expected to stabilize Mo atoms, potentially mitigating degradation processes associated with the formation of oxygen vacancies [37]. In comparison, PBtA reduces charge transfer, resulting in a weaker electronic stabilization effect.

### 2.4 Device‐Level Verification of the PBN Passivation Effect

**FIGURE 3:**
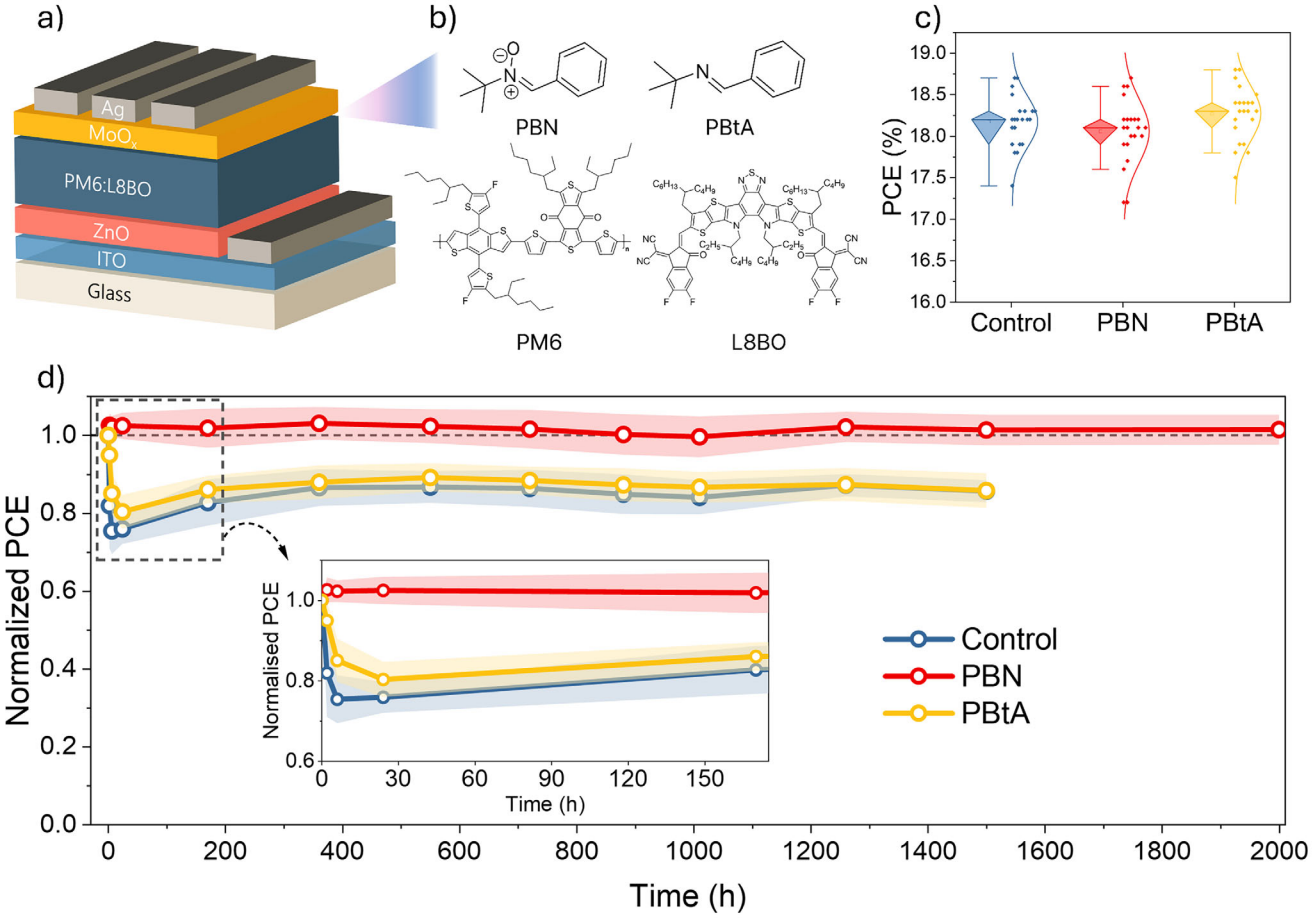
(a) Device structure of I‐OSCs and (b) chemical structure of the PBN, PBtA, PM6, and L8BO. (c) Diamond box plots illustrating the deviation distribution of the PCE for Control, PBN, and PBtA devices, obtained from over 20 devices each. (d) PCE evolution of the Control, PBN, and PBtA devices under continuous thermal stress at 85°C in an inert atmosphere. The inset shows a magnified view of the PCE changes during the initial 170 h of aging. The data points represent the average values, and the shaded regions correspond to the standard deviation calculated from over 10 devices. The initial average PCE values for the Control, PBN, and PBtA devices were 17.4%, 17.1%, and 17.2%, respectively.

To verify the device‐level impact of the PBN passivation, we fabricated I‐OPVs (ITO/ZnO/PM6:L8BO/bare, PBN‐, or PBtA‐treated MoO_x_/Ag) (Figure 3a,b) for thermal aging studies. All three devices exhibited nearly identical initial PCEs (∼18.1–18.3% on average; Figure 3c), confirming that the thin PBN and PBtA interlayers did not compromise initial device performance. After thermal aging at 85°C (Figure 3d), the Control device exhibited a rapid PCE drop during the initial aging stage—commonly referred to as ‘burn‐in loss’—with its PCE decreasing by 20% within 2 h and by 25% after 24 h. The PBtA device showed a delayed PCE drop but ultimately reached a similar degradation level as the Control device after 24 h. In contrast, the PBN device demonstrated remarkable thermal stability, maintaining 100% of its initial PCE for over 2000 h with no burn‐in loss.

Table 1 summarizes the evolution of the individual photovoltaic parameters during the early burn‐in regime and at a later aging time (fresh, 2, 6, 24, and 165 h), highlighting different degradation pathways in the three devices. The corresponding current density–voltage (*J–V*) curves and external quantum efficiency (EQE) spectra for all devices are provided in Figures S6 and S7. For the Control device, the rapid PCE loss (0–24 h) is primarily driven by a steep drop in short‐circuit current density (J_SC_) from 26.7 to 22.9 mA cm^−2^. However, at an extended aging time (165 h), while the fill factor and open‐circuit voltage (V_OC_) continued to decrease slightly, J_SC_ unexpectedly recovered to nearly its initial value (26.7 mA cm^−^^2^ at 165 h). A similar non‐monotonic trend of J_SC_ has been reported recently in I‐OSCs under accelerated thermal aging conditions (150°C for 30 min) [21]. Notably, this result contradicts the expected outcome from the XPS data (Figure 1c), which showed a continuous decrease in the WF of MoO_x_ from 24 to 165 h.

**TABLE 1:** Photovoltaic performance summary of the Control, PBN, and PBtA devices during thermal aging at 85°C.^a^

Thermal aging time	Device	*V*_OC_ (V)	*J*_SC_ (mA cm^−2^)	*FF* (%)	PCE (%)
Fresh	Control	0.902 ± 0.004 (0.904)	26.7 ± 0.5 (27.6)	75.8 ± 0.6 (75.2)	18.2 ± 0.3 (18.8)
	PBN	0.894 ± 0.004 (0.891)	26.7 ± 0.4 (27.6)	75.6 ± 0.5 (75.1)	18.1 ± 0.2 (18.5)
	PBtA	0.901 ± 0.003 (0.898)	26.8 ± 0.6 (27.5)	75.5 ± 0.6 (75.2)	18.2 ± 0.3 (18.6)
2 h	Control	0.916 ± 0.000 (0.916)	23.3 ± 0.5 (23.8)	74.1 ± 0.5 (75.1)	15.8 ± 0.4 (16.3)
	PBN	0.899 ± 0.003 (0.898)	27.0 ± 0.5 (27.8)	75.7 ± 0.4 (75.6)	18.3 ± 0.3 (18.9)
	PBtA	0.914 ± 0.003 (0.910)	24.7 ± 0.6 (25.2)	75.0 ± 0.4 (75.5)	16.9 ± 0.4 (17.3)
6 h	Control	0.908 ± 0.003 (0.910)	22.1 ± 0.4 (22.4)	70.0 ± 0.6 (70.7)	14.0 ± 0.3 (14.4)
	PBN	0.891 ± 0.000 (0.891)	27.3 ± 0.5 (28.1)	74.9 ± 0.6 (74.6)	18.2 ± 0.3 (18.7)
	PBtA	0.909 ± 0.003 (0.910)	23.1 ± 0.6 (23.5)	71.2 ± 0.5 (71.3)	14.9 ± 0.4 (15.2)
24 h	Control	0.874 ± 0.005 (0.873)	22.9 ± 0.4 (23.0)	68.0 ± 0.5 (68.1)	13.7 ± 0.3 (13.7)
	PBN	0.883 ± 0.003 (0.879)	27.4 ± 0.5 (28.2)	74.8 ± 0.6 (74.4)	18.1 ± 0.2 (18.4)
	PBtA	0.882 ± 0.003 (0.885)	22.7 ± 0.6 (23.4)	68.2 ± 0.3 (68.3)	13.7 ± 0.4 (14.2)
165 h	Control	0.822 ± 0.011 (0.813)	26.7 ± 0.3 (26.7)	65.4 ± 1.2 (66.9)	14.4 ± 0.3 (14.5)
	PBN	0.880 ± 0.003 (0.879)	28.3 ± 0.5 (28.7)	72.7 ± 0.4 (72.2)	18.1 ± 0.2 (18.2)
	PBtA	0.819 ± 0.008 (0.831)	26.3 ± 0.5 (27.0)	65.3 ± 1.1 (66.0)	14.1 ± 0.6 (14.8)

^a^ The values are presented as average ± standard deviation from five devices; values in parentheses correspond to the specific device that exhibited the best initial performance in the fresh state, tracked throughout the aging process.

### 2.5 Spatially Resolved Analysis of MoO_x_ Diffusion Dynamics

As J_SC_ recovery cannot be explained by interfacial WF degradation alone (Figure 1c), it is more likely attributed to the dynamic evolution of the charge‐carrier distribution within the BHJ layer, driven by the thermally activated diffusion of MoO_x_ species, as depicted in Scheme 1. Previous capacitance‐based study has demonstrated that diffused MoO_x_ species can act as both p‐dopants and trap sites in the BHJ layer [22]. Thus, we employed drive‐level capacitance profiling (DLCP) to extract carrier density (*N*) as a function of profiling depth (*<x>*), analyzing the non‐linear capacitance response to varying AC drive levels (*δV*) (detailed principles in the Supporting Information). For this analysis, the ZnO/BHJ interface was characterized as an n^+^–p junction, as detailed in Figure S8.

**FIGURE 4:**
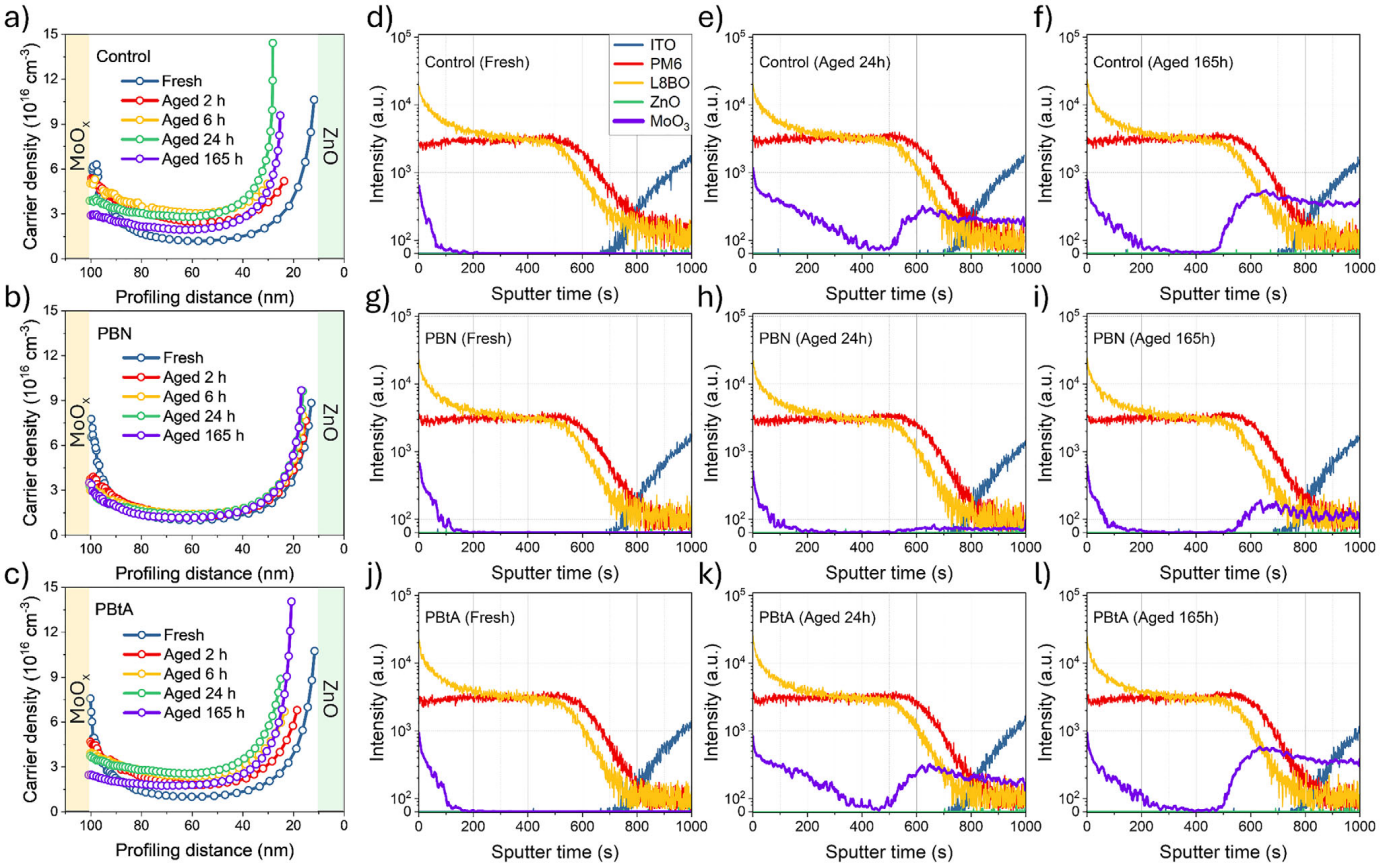
Evolution of carrier density profiles for (a) Control, (b) PBN, and (c) PBtA devices during thermal aging at 85°C, as measured by DLCP. OrbiSIMS negative ion depth profiles of the ITO/ZnO/BHJ stack after mechanical delamination of the Ag/MoO_x_ layers for (d–f) Control, (g–i) PBN, and (j–l) PBtA devices in the fresh state and after aging at 24 and 165 h.

Figure 4a–c displays the carrier density profiles for the three device configurations at various stages of thermal aging. For the Control device, the carrier density within the BHJ layer increased substantially during the first 24 h of aging except near the MoO_x_ interface. At the representative bulk position of 60 nm, the carrier density increased from ∼1.2 × 10^16^ cm^−3^ (fresh) to ∼2.8 × 10^16^ cm^−3^ (24 h). Concurrently, an electrically inactive region emerged near the ZnO/BHJ interface, as indicated by a shift in the profiling onset (minimum profiling distance) toward the bulk. However, after aging for 165 h, the carrier density exhibited reversal behavior, declining to ∼1.9 × 10^16^ cm^−3^ below the 24 h level, while the thickness of the electrically inactive region also decreased. This variation in bulk carrier density aligns with the J_SC_ trend observed in Table 1. The PBtA device showed a similar evolution of carrier density and electrically inactive regions as the Control device, although the changes were slightly less pronounced. Unlike the other devices, the PBN device (Figure 4b) exhibited minimal changes in its DLCP profiles throughout the aging process, consistent with its nearly unchanged J_SC_.

To identify the chemical origin of the dynamic charge‐carrier distribution in the BHJ layer, we performed orbital trap secondary ion mass spectrometry (OrbiSIMS) depth profiling on devices aged for different durations (fresh, 24, and 165 h). To ensure depth‐profile accuracy and to minimize artifacts such as beam‐induced knock‐on mixing from the top layers, the Ag/MoO_x_ layers were mechanically removed with adhesive tape prior to analysis, and sputtering was performed from the exposed BHJ surface downward (Figure 4d–l). The MoO_3_^−^, C_10_S_2_^−^, C_84_H_90_F_4_N_8_O_2_S_5_^−^, ZnO_7_H^−^, and InO_2_^−^ ions were assigned to MoO_x_, PM6, L8BO, ZnO, and ITO, respectively (Figure S9). In the fresh state, all the devices exhibited well‐defined profiles, with the MoO_x_ signal confined near the top surface, likely originating from species that diffused into the BHJ layer during MoO_3_ deposition. Upon thermal aging, pronounced MoO_x_ migration was observed in both the Control and PBtA devices. After 24 h (Figure 4e,k), the MoO_3_^−^ signal broadened substantially, penetrating from the top surface deep into the BHJ bulk and accumulating at the BHJ/ZnO interface. Notably, at 165 h (Figure 4f,l), the MoO_3_^−^ signals in the BHJ bulk layer decreased markedly, while further accumulation occurred at the BHJ/ZnO interface, indicating a two‐stage redistribution—initial diffusion into the bulk (0–24 h), followed by progressive migration and accumulation toward the ZnO interface (24–165 h).

As previously reported, MoO_x_ species act as both p‐dopants and trap sites in organic semiconductors owing to their high electron affinity [38]. When accumulated at the interfacial region, the resulting p‐doping and high trap density may pin the Fermi level within the interfacial region, making the local energy bands insensitive to applied AC/DC biases [39]. Consequently, this pinned region does not contribute to the DLCP signal, manifesting as an electrically inactive region that shifts the effective profiling onset away from the interface, consistent with the DLCP observations. In stark contrast, the PBN device (Figure 4g–i) exhibited markedly suppressed MoO_x_ diffusion. The MoO_3_^−^ signal remained tightly confined near the top surface at 24 h, with minimal accumulation at the BHJ/ZnO interface, even after 165 h of thermal stress. These results provide direct evidence that PBN effectively immobilizes MoO_x_ at the interface and prevents the redistribution observed in the Control and PBtA devices.

### 2.6 Charge Dynamics Analysis and Drift‐Diffusion Modeling

**FIGURE 5:**
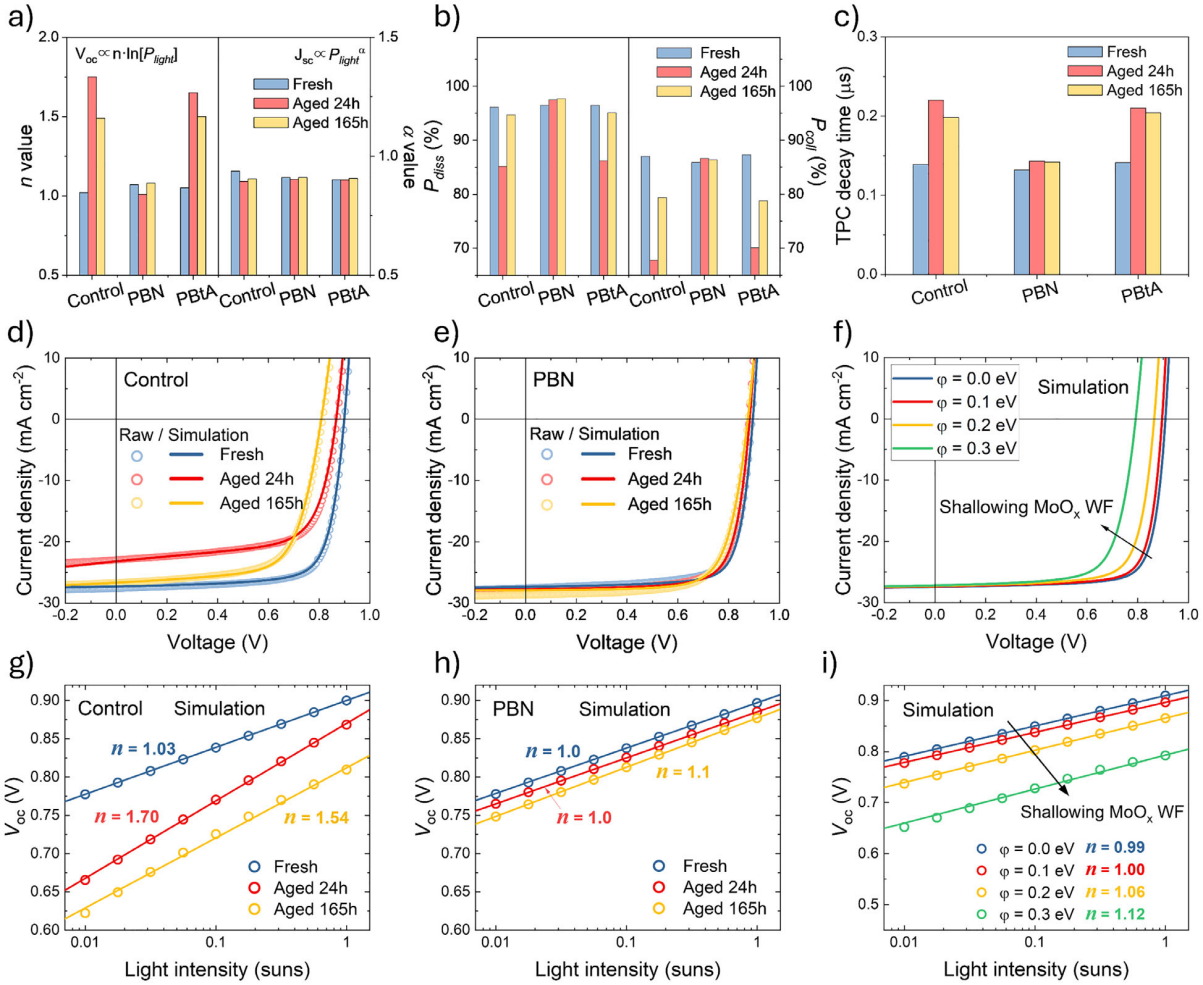
(a–c) Comparison of Control, PBN, and PBtA devices at fresh, 24 h, and 165 h aging: (a) ideality factor (n) and exponent alpha (α) derived from light intensity‐dependent V_OC_ and J_SC_ measurements, (b) exciton dissociation (P_diss_) and charge collection (P_coll_) probabilities, (c) average transient photocurrent (TPC) decay times. (d,e) Experimental (symbols) and drift‐diffusion simulated (lines) *J–V* curves for (d) Control and (e) PBN devices at different aging stages. (g,h) Experimental (symbols) and simulated (lines) V_OC_ vs. light‐intensity plots with extracted ideality factors for (g) Control and (h) PBN devices. (f,i) Effect of simulated MoO_x_ work function (WF) shallowing (Φ = 0.1−0.3 eV) on (f) *J–V* characteristics and (i) V_OC_ vs. light intensity, compared with an ideal interface (Φ = 0.0 eV).

To elucidate how spatially distributed MoO_x_ species modulate photovoltaic degradation, we comprehensively investigated charge‐carrier dynamics and electro‐optical drift‐diffusion modeling. First, the light intensity (*P_light_*) dependence of the J_SC_ and V_OC_ was measured to reveal the recombination behavior (Figure 5a and Figure S10; detailed values in Table S1). The ideality factor *n* (derived from *V_OC_* ∝*n*(*kT*/*q*) *ln*(*P_light_*)) increased after thermal aging for the Control and PBtA devices, from ∼1.0 (fresh) to 1.75 (Control) and 1.65 (PBtA) at 24 h, indicating enhanced trap‐assisted recombination. At 165 h, *n* partially decreased (Control: 1.49; PBtA: 1.50), consistent with MoO_x_ migration from the bulk to the interface that reduces trap sites in the bulk region. In contrast, the PBN device maintained *n* ≈ 1.0 throughout the aging process, confirming effective trap suppression. Meanwhile, the light‐intensity exponent α (fitted from $J_{SC}\propto P_{light}^{\alpha }$) remained ∼0.90 for all devices and aging times, indicating that bimolecular recombination is not the dominant degradation pathway.

To understand how the MoO_x_ distribution affects charge extraction, effective voltage (V_eff_) and transient photocurrent (TPC) were measured (Figure 5b,c, Figures S11 and S12; detailed values are provided in Tables S2 and S3). For the Control and PBtA devices, both the exciton dissociation (*P_diss_*) and charge collection (*P_coll_*) probabilities decreased sharply from ∼96% to 85%–86% and ∼87% to 67%–70% after 24 h of aging, respectively. After 165 h, *P_diss_* nearly recovered to ∼94%–95%, whereas *P_coll_* only reached 78%–79%, indicating that charge collection remains the limiting factor despite the recovery of exciton dissociation. We note that while reverse‐bias collection losses can affect the absolute values of *P_diss_* and *P_coll_*, the relative trends and differences across devices remain clear. Combined with prior MoO_x_ redistribution data, these trends suggest that the diffused MoO_x_ species in the BHJ bulk layer suppress exciton dissociation, whereas the accumulated MoO_x_ species at the cathode side limit charge collection. In contrast, the PBN device maintained both *P_diss_* (96%–97%) and *P_coll_* (85%–86%) throughout the aging process, confirming the suppression of both degradation pathways.

TPC measurements further elucidate the charge extraction dynamics (Figure 5c). For the Control device, the average photocurrent decay time (τ_avg_) increased from 0.14 µs (fresh) to 0.22 µs at 24 h (58% slower), subsequently recovering to 0.20 µs at 165 h (42% slower than fresh). The PBtA device also exhibited similar behavior. This dynamic feature confirms that both the bulk‐diffused and interface‐accumulated MoO_x_ species suppress extraction rates. Conversely, the PBN device maintained a nearly constant τ_avg_ (0.13 µs → 0.14 µs), indicating that it preserves efficient extraction kinetics.

To validate the experimental observations and elucidate the degradation mechanism, we performed electro‐optical drift‐diffusion modeling, adopting the approach reported by Xi et al. [21]. (Figure 5d–i and Figure S13 for energy level diagrams; Tables S4 and S5 for simulation parameters). This model accurately reproduced the experimental trends linking the MoO_x_ impurity distribution with device performance parameters (refer to the Supporting Information for details). For the Control device, the simulation matched the experimental *J–V* curve at 24 h by assuming bulk‐distributed MoO_x_ impurities, which induced unintentional p‐doping and enhanced trap‐assisted recombination, fully accounting for the observed J_SC_ loss. Concurrently, this model revealed impurity accumulation at the ZnO interface, creating a space charge in a few‐nm‐thick regions. The space charge induced strong band bending that acted as an injection barrier, accounting for the reduced V_OC_. The combined bulk and interfacial accumulation precisely reproduced the high ideality factor (*n* = 1.70), consistent with our light‐intensity measurements.

Crucially, the 165 h‐aged Control device was simulated by modeling the migration of these bulk impurities to the ZnO interface. This substantial reduction of impurities from the bulk layer reduces the trap‐assisted recombination. Furthermore, such impurity accumulation near the ZnO interface induces a strong local electric field that facilitates charge carrier extraction. This field‐assisted extraction likely contributes to the near‐complete recovery of J_SC_, compensating for the residual transport losses imposed by the remaining bulk impurities that were detected via DLCP. However, on the downside, this intensified impurity accumulation at the ZnO interface simultaneously exacerbated the local band bending and created a stronger non‐ohmic barrier. This counter‐effect precisely accounts for the further V_OC_ reduction and the persistently high ideality factor (*n* = 1.54), which confirms that the recombination occurred mainly at the cathode interface.

PBN device simulations also reproduced the experimental data: no J_SC_ loss, minimal V_OC_ reduction, and stable *n* ≈ 1 (Figure 5e,h). The model could even capture the slight J_SC_ enhancement observed in the aged PBN device (Table 1). This is similarly attributed to the minor accumulation of MoO_x_ at the ZnO interface, which strengthens the local electric field and aids carrier extraction. This effect may partially compensate for the performance losses induced by other minor aging factors, such as interfacial chemical degradation and subtle morphological changes, thereby contributing to the negligible burn‐in loss observed in the PBN device. Collectively, these findings confirm that PBN effectively suppresses MoO_x_ diffusion, thereby preventing the formation of bulk and interfacial traps.

Importantly, the observed V_OC_ loss cannot be attributed solely to the MoO_x_ WF reduction (0.09–0.12 eV; Figure 1c). Simulations incorporating a 0.1 eV WF shift as an energy‐level offset at the BHJ/MoO_x_ interface showed negligible impacts on the photovoltaic parameters and ideality factors (Figure 5f,i), inconsistent with the experimental results. Even assuming a larger WF shift (0.2–0.3 eV), the simulated ideality factor remained near unity (*n* ≈ 1.1), showing a notable discrepancy with the experimental value (*n* ≈ 1.5). Consequently, the simulations indicated that impurity‐induced recombination and band bending at the ZnO interface are the dominant drivers of degradation.

### 2.7 Generality of PBN Passivation Across Different Acceptor Systems

**FIGURE 6:**
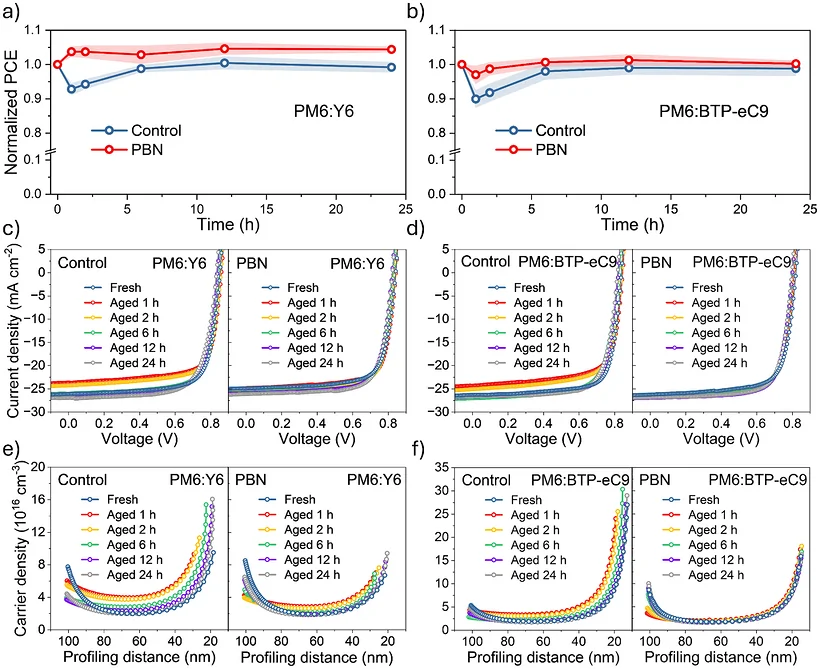
Generality of the PBN passivation strategy for enhancing thermal stability across different BHJ systems. (a,b) Normalized PCE evolution of Control and PBN devices based on (a) PM6:Y6 and (b) PM6:BTP‐eC9 during thermal aging at 85°C for 24 h. Lines represent average PCE, and shaded areas indicate standard deviation from three devices. (c,d) Evolution of *J–V* curves for (c) PM6:Y6 and (d) PM6:BTP‐eC9 devices measured at different aging times (fresh, 1, 2, 6, 12, 24 h). (e,f) Corresponding carrier density profiles measured by DLCP for (e) PM6:Y6 and (f) PM6:BTP‐eC9 devices.

To assess the broader applicability, we evaluated the efficacy of the PBN passivation strategy in PM6:Y6 and PM6:BTP‐eC9 devices under identical thermal stress (85°C). PBN almost maintained the initial PCEs throughout 24 h (Figure 6a,b), while the Control devices showed rapid degradation within 6 h, followed by partial recovery. *J–V* and DLCP (Figure 6c–f) confirmed that performance loss and recovery can be attributed to J_SC_ changes coupled with MoO_x_ redistribution, which aligns with the mechanism established in PM6:L8BO. These results demonstrate that PBN passivation is a universally effective strategy for suppressing MoO_x_‐induced degradation, irrespective of the NFA molecular structure.

Closer examination reveals a distinct difference in the degradation kinetics. PM6:L8BO exhibited slow, continuous MoO_x_ diffusion over days, whereas the PM6:Y6/BTP‐eC9 systems showed accelerated diffusion within 1–2 h, accompanied by a rapid bulk carrier density elevation and the formation of an electrically inactive region near the ZnO interface (Figure 6e,f). Critically, this MoO_x_ distribution markedly dissipated at 24 h, the bulk carrier density returned to its initial values, and the inactive region vanished. Correspondingly, J_SC_ recovery was accompanied by minimal V_OC_ loss, revealing that the MoO_x_ accumulation became transient in these faster‐diffusion BHJ systems, whereas it remained irreversible in PM6:L8BO. This kinetic difference may be attributed to the fundamental variations in molecular packing, which is controlled by the alkyl chain topology of the NFAs. Li et al. [40] reported that Y6 exhibited a substantially lower packing coefficient (54.5%) with larger orthorhombic packing voids, whereas L8BO showed a higher packing coefficient (64.1%) with smaller ellipse‐shaped packing voids, indicative of a more condensed molecular packing motif in single‐crystal structures. The same study further showed that L8BO effectively preserves its packing characteristics in thin films. Therefore, we speculate that the condensed molecular packing associated with the branched alkyl‐chain topology of L8BO provides a more restrictive molecular environment, which effectively traps the MoO_x_ species kinetically. In contrast, the relatively less condensed molecular packing associated with the linear alkyl‐chain topology of Y6 and BTP‐eC9 may contribute to the faster MoO_x_ redistribution and the subsequent thinning of the accumulated region, thereby restoring efficient carrier extraction. However, direct structural characterization of the actual blend films would be required to establish this relationship conclusively.

Furthermore, to define the scope of this interface engineering strategy, we additionally examined the photostability of the Control, PBN‐, and PBtA‐treated PM6:L8BO devices under continuous 1‐sun illumination in a nitrogen atmosphere (Figure S14). All three devices exhibited nearly identical degradation behavior, retaining ∼50% of their initial PCE after 100 h of light soaking. This result suggests that the stabilizing effect of PBN is relevant to thermally induced degradation at the MoO_x_ interface, whereas the instability under illumination likely arises mainly from degradation pathways associated with the ZnO/BHJ interface. This interpretation is consistent with previous literature showing that the UV‐activated photocatalytic effect of ZnO can trigger the degradation of NFAs, thereby limiting the photostability of I‐OSCs [41, 42].

## 3 Conclusions

This work demonstrates a robust and facile nitrone‐based interface engineering strategy for achieving thermally stable MoO_x_‐based I‐OSCs. The incorporation of PBN enables its highly polar nitrone moiety to strongly passivate under‐coordinated Mo sites, suppressing oxygen vacancy formation under thermal stress. Control experiments using an imine analogue (PBtA) verify the critical role of nitrone in interface stabilization. Comprehensive characterization and modeling confirm that MoO_x_ diffusion and redistribution within the BHJ layer dominate the device‐level thermal degradation rather than the WF reduction of MoO_x_. PBN suppresses MoO_x_ diffusion into the BHJ layer, mitigates diffusion‐induced trap formation, and preserves efficient charge extraction. This enables PM6:L8BO devices to retain ∼100% of initial efficiency (∼18.1%) after 2000 h at 85°C without burn‐in loss. The effectiveness of this strategy is further validated in the PM6:Y6 and PM6:BTP‐eC9 systems. This study provides insights into MoO_x_‐mediated degradation and presents a practical solution‐processable route to develop long‐term stable I‐OSCs.

## 4 Experimental Section

### 4.1 Materials

PBN and PBtA were purchased from Sigma–Aldrich and Angene Chemical, respectively. 1,8‐diiodooctane (DIO) and 1‐Chloronaphthalene (CN) were purchased from Tokyo Chemical Industry Co., Ltd. Diiodomethane (DIM), Chloroform (CF), ethanolamine (MEA), isopropyl alcohol (IPA), and zinc acetate dehydrate (ZAD) were purchased from Sigma–Aldrich. PM6, L8BO, Y6, and BTP‐eC9 were purchased from Solarmer Materials Inc.

### 4.2 Solution Preparation

ZnO sol–gel solution was prepared by dissolving 405 mg of ZAD in 16.4 mL of IPA and then adding 200 mg of MEA. PM6:L8BO (D:A = 1:1.2, by weight) was dissolved in CF with a concentration of 17.6 mg/mL and adding 0.3 vol% DIM as an additive. PM6:Y6 (D:A = 1:1.2, by weight) and PM6:BTP‐eC9 (D:A = 1:1.2, by weight) were dissolved in CF with a concentration of 16.5 mg/mL, and adding 0.5 vol% CN and 0.5 vol% DIO as additives, respectively. For the passivation solutions, PBN and PBtA were dissolved in IPA with a concentration of 1 mg/mL, respectively. This concentration was selected based on preliminary concentration‐screening experiments, in which 1 mg mL^−1^ provided the highest average device performance within the tested range (Figure S15)

### 4.3 Device Fabrication

The OSC devices were fabricated with an inverted configuration of glass/ITO/ZnO/BHJ layer/MoO_3_/Ag. The ITO glass substrates were sonicated sequentially in DI water, acetone, and IPA, followed by drying in an oven. The substrates were treated in a UVO chamber for 20 min before use. ZnO solution was coated on the substrate via spin‐coating at 5000 rpm for 20 s, and then annealed at 200°C for 10 min on a hotplate in the air condition. BHJ solutions (17 µL) were dynamically coated onto the ZnO layer in a N_2_‐filled glove box, yielding films with a thickness of approximately 100 nm, and then the films were annealed at 100°C for 5 min. Subsequently, for PBN or PBtA treatment, each solution was spin‐cast at 5000 rpm for 30 s on the BHJ films. Finally, thermal evaporation under 2 × 10^−6^ Torr was employed to deposit MoO_3_ (5 nm) and Ag (100 nm) onto the BHJ layers via shadow masking, producing devices with active areas of 4.64 mm^2^.

### 4.4 Characterization

#### 4.4.1 Photovoltaic Performance Analysis

The current density–voltage (*J–V*) characteristics were measured using a Keithley 2420 Source/Measure unit under AM 1.5 G solar illumination (100 mW/cm^2^; Oriel Sol3A 450 W, Newport). Forward voltage scans were conducted from −0.2 to 1.0 V. Light intensity calibration was performed using a KG1‐filtered silicon reference cell (Newport Calibration). External quantum efficiency (EQE) spectra were acquired using an ORIEL QuantX 300 (Newport Corp.) calibrated with a Newport‐standardized reference detector.

#### 4.4.2 XPS Measurement

X‐ray Photoelectron Spectroscopy (XPS) analysis was conducted using a Nexsa system (ThermoFisher Scientific, Inc.). The system was equipped with a monochromatic Al‐Kα x‐ray source. To prevent surface contamination by ambient air exposure, samples were prepared inside a N_2_‐filled glovebox. The stacks of MoO_x_/Ag were mechanically delaminated using adhesive tape and subsequently transferred immediately to the analysis chamber using a high‐vacuum transfer module. To exclude batch‐to‐batch variation in the MoO_x_ deposition, all MoO_x_ layers used for the 9 samples (Control, PBN, and PBtA; fresh, 24 h, and 165 h for each condition) were fabricated in a single deposition batch.

#### 4.4.3 FTIR Spectroscopy

Fourier‐transform infrared (FTIR) analysis was conducted using a Vertex 70v spectrometer (Bruker) in transmission mode. The spectra were acquired with a high spectral resolution of 1 cm^−^^1^ and accumulated over 60 scans. Samples were prepared on potassium bromide (KBr) discs in the form of either a pristine PBN or a MoO_x_ (10 nm)/PBN structure.

#### 4.4.4 OrbiSIMS Depth Profiling

OrbiSIMS depth profiling was conducted using an M6 hybrid SIMS instrument (IONTOF GmbH, Münster, Germany) located at the Korea Basic Science Institute (KBSI) Busan Center, following the methodology established by Passarelli et al. [43]. The main analysis chamber was maintained at a base pressure of 1.0 × 10^−7^ mbar. For the depth analysis, a 20‐keV Ar2043+ gas cluster ion beam (GCIB) was employed as the sputter source, operating in a long pulse mode. This beam was defocused to 20 µm and operated with a 110 pA current, a 200 µs cycle time, and a 1.0% duty cycle. All secondary ion mass spectra were acquired in negative polarity mode. Data was collected from a 300 µm^2^ analysis area using a random raster pattern, centered within a larger 420 µm^2^ sputter crater.

#### 4.4.5 DLCP Measurement

DLCP measurements were performed using a variable AC voltage (V_ac_) superimposed onto a DC bias (V_dc_), following previously reported procedures [44]. The AC signal had a fixed frequency of 10 kHz and an amplitude that varied from 50 to 200 mV. The DC bias was swept from 0 V to the device's open‐circuit voltage (V_OC_, e.g., 0.9 V). Critically, for each V_ac_ amplitude, an additional DC offset was applied to ensure the maximum forward bias remained constant throughout the measurement.

The non‐linear capacitance response (dQ/dV) as a function of the applied AC voltage (dV) is modeled by the following polynomial expansion:

$$\frac{dQ}{dV}=C_{0}+C_{1}dV+C_{2}{\left(dV\right)}^{2}+\cdots$$

By fitting the experimental capacitance data to this expression, we extracted the C_0_, which represents the capacitance value at the limit of zero AC amplitude, and the first‐order coefficient (C_1_). These parameters allow for the calculation of the carrier density (N) and the corresponding profiling depth (< x >) from the junction using the relations:

$$N=-\;\frac{C_{0}^{3}}{2q\varepsilon A^{2}C_{1}}$$

$$\langle x\rangle =\frac{\varepsilon A}{C_{0}}$$

where q is the elementary charge, ε is the dielectric constant of the semiconductor, and A is the active area of the device. A spatial profile of the carrier density (N(<x>)) was then obtained by plotting the calculated N values against the profiling depth <x>, which is tuned by varying the applied V_dc_.

#### 4.4.6 TPC measurement

A pump wavelength of 470 nm and intensity of < 0.01 µJ/cm^2^ were used. The time decay constants were obtained by deconvoluting the measured signal decay from the pump beam time profile (characterized by a full‐width‐at‐half‐maximum of ∼200 ps) and fitting to a sum of the exponential terms, i.e., ΔI(t) = A_1_ exp(−t/τ_1_) + A_2_ exp(−t/τ_2_), where ΔI(t) is the time‐dependent transient photocurrent intensity, A is the amplitude (normalized percentage [A_i_ / (|A_1_|+|A_2_|) × 100]), and τ is the fitted decay time. The averaged decay times (τ_avg_) were obtained using (A_1_τ_1_ + A_2_τ_2_)/(A_1_ + A_2_). The fitted decay parameters for all samples are summarized in Table S3.

### 4.5 Computational Details

#### 4.5.1 DFT Calculation for Isolated Molecules

Density functional theory (DFT) calculations were performed using the Gaussian 16 package [45]. Geometry optimizations of isolated conformers of PBN and PBtA were conducted at the ωB97X‐D/6‐311++G(d,p) level of theory [46]. The ωB97X‐D functional, which incorporates long‐range exact exchange and dispersion corrections, was selected due to its reliable performance in describing molecular electrostatics and weak intermolecular interactions. Electrostatic potential (ESP) surfaces of the optimized structures were visualized using GaussView and quantitatively analyzed with Multiwfn to determine the distribution and extrema of the surface electrostatic potential [47].

#### 4.5.2 DFT Calculation for MoO_x_ Surface Interaction

To investigate the interaction between the molecules and the oxide surface, we carried out DFT calculations for MoO_3_, MoO_3_–PBN, and MoO_3_–PBtA systems using the Vienna Ab initio Simulation Package (VASP). The computational study was initiated by constructing the MoO_3_ (111) surface structure. Oxygen vacancy sites were introduced to model the defective surface, followed by the generation of PBN‐ and PBtA‐substituted models where the molecules occupy the oxygen vacancy sites. The electronic structures were calculated using the frozen‐core projector augmented wave (PAW) method [48, 49]. The exchange‐correlation interaction was described by the generalized gradient approximation (GGA) [50, 51, 52] with the Perdew‐Burke‐Ernzerhof (PBE) functional [53]. A plane‐wave cutoff energy of 500 eV was employed, and the irreducible Brillouin zone was sampled using a Monkhorst‐Pack k‐point mesh corresponding to 20 k‐points. Finally, the Bader charge analysis was performed to evaluate the charge redistribution induced by oxygen vacancy formation and molecular substitution [54].

#### 4.5.3 Electro‐Optical Device Simulations

For the device simulations, a combined electro‐optical device model was used [55]. Herein, the optical behavior is described by an optical transfer‐matrix model which accounts for the wavelength‐dependent reflection, absorption, and interference of light in the device stack [56]. The refractive indices and extinction coefficients of the different layers constituting the device stack are used as input to calculate the generation rate of electron‐hole pairs inside the active layer for every wavelength, assuming an incident light intensity spectrum given by AM1.5G. The calculated photogeneration rate, given as a function of the position x within the active layer, is then used as input for the electrical model. Electrical behavior is described using a drift‐diffusion model, which accounts for transport, recombination, and space charge effects of charges within the active layer. The active layer is described as an effective semiconductor where the transport of electrons occurs in the acceptor LUMO level and holes in the donor HOMO level. The model numerically solves the coupled Poisson equation, the charge carrier continuity equations, drift‐diffusion equations for the electrical potential, electron density, and hole density [57]. The electron transport layer (ZnO) and the hole transport layer (MoO_x_) layers are treated as part of the cathode and anode electrodes, respectively, with the cathode contact at x = 0 and anode contact at x = d, where d is the thickness of the active layer. At the contacts, thermal equilibrium conditions are assumed to apply. The recombination of charge carriers in the active layer can take place either via bimolecular recombination or by trap‐assisted Shockley‐Read‐Hall (SRH) recombination via MoO_x_ impurity‐induced traps. The MoO_x_ impurities are assumed to act as deep traps having a step‐like spatial distribution with a trap density given by N_t, tot_ = N_t, bulk_ + N_t, int_(x), consisting of a bulk trap density N_t, bulk_ distributed uniformly throughout the active layer and an additional trap density N_t, int_(x) accumulated near the cathode (ZnO) interface. Here, N_t, int_(x) = N_t, int_ for x < d_int_, while N_t, int_(x) = 0 otherwise, where d_int_ is the width of the interfacial accumulation region. We assume d_int_ = 8 nm for the 24 h aged control device and d_int_ = 4 nm for the other devices. The MoO_x_ impurity‐induced trap states are assumed to be acceptor‐type electron traps being negatively charged when occupied by an electron (effectively an ionized p‐dopant) or neutral when unoccupied (i.e., occupied by a hole). Finally, transport losses within the charge carrier transport layers, the electrodes, and the external circuitry, are accounted for by an added external series resistance R_series_ (given in units of Ω cm^2^). The parameters used for the simulations are given in Tables S4 and S5.

## Funding

This work was financially supported by the Korea Institute of Energy Technology Evaluation and Planning (KETEP) and the Ministry of Trade, Industry & Energy (MOTIE) of the Republic of Korea (No. RS‐2024‐00457537). This research was also supported by the National Research Foundation of Korea (NRF) grants funded by the Ministry of Science and ICT (MSIT) (No. RS‐2024‐00349326, No. RS‐2025‐00514103, and No. RS‐2022‐NR068223) and the Ministry of Education (LAMP Program, No. RS‐2024‐00442775). In addition, this work was supported by the Technology Development Program (No. RS‐2023‐00282868) funded by the Ministry of SMEs and Startups (MSS), Korea, and by the GIST ISD program of the Ministry of Science and ICT (No. KH1150). O.J.S. acknowledges funding from the Research Council of Finland through Project No. 357196.

## Conflicts of Interest

The authors declare no conflicts of interest.

## Supporting information




**Supporting File**: smll74851‐sup‐0001‐SuppMat.docx.

## Data Availability

The data that support the findings of this study are available from the corresponding author upon reasonable request.
