# Supplementary material for: Burn‐in‐Loss‐Free Organic Solar Cells With Over 2000 h of Thermal Stability Through Nitrone‐Based Interface Passivation
Source: Small. 2026 Jul 31;22(54):e74851. doi: 10.1002/smll.74851 (PMC13614277; doi:10.1002/smll.74851)
Supplement: Supplementary file 1 [file SMLL-22-e74851-s001.docx]

Supporting Information

Burn-in-Loss-Free Organic Solar Cells with Over 2,000 h of Thermal Stability through Nitrone-Based Interface Passivation

Sanseong Lee, Kiyoung Park, Changhoon Lee, Oskar J. Sandberg, Ju-Hyeon Kim, Taeyoon Ki, Jong-Hoon Lee, Dongguen Shin, Jong Sung Jin, Ji Yeong Sung, In-Wook Hwang, Jubin Jang, Kitae Kim, Soohyung Park, Ji Hoon Shim, Hongkyu Kang* and Kwanghee Lee*

**Figure S1** Photograph of the vacuum transfer chamber used for XPS analysis. Samples, mechanically delaminated in a N_2_-filled glovebox, were immediately transferred to the XPS system using this chamber to prevent air exposure and surface contamination.

**Figure S2** FTIR spectra of pristine PBN and MoO_x_/PBN samples.

**Figure S3** Mo‑projected DOS for pristine MoO_3_, oxygen‑deficient MoO_x_, and passivated MoO_x_/PBN and MoO_x_/PBtA, showing vacancy‑induced mid‑gap states in MoO_x_ that are strongly suppressed upon passivation. The inset enlarges 0.5–2.2 eV to highlight these states and an additional peak in MoO_x_/PBN (asterisk), originating from the PBN molecule rather than a defect state.

**Figure S4** Schematic of the PBN-passivated MoO_x_ surface, highlighting the six surface oxygen sites (labeled 1–6) at which oxygen atoms were removed to evaluate the OVFE. The same sites were analyzed for the MoO_3_, MoO_x_, and PBtA-passivated MoO_x_ systems.

**Figure S5** Bader charge redistribution upon PBN passivation of oxygen‑deficient MoOₓ, highlighting electron transfer from reduced molybdenum sites into the adsorbed PBN molecule and delocalization of excess electrons across its phenyl–nitrone core.

**Figure S6** J–V curves of (a) Control, (b) PBN, and (c) PBtA devices measured at different aging times (fresh, 2, 6, 24, and 165 h) under continuous thermal stress at 85 °C in N₂ atmosphere.

**Figure S7** EQE spectra (open circles) and integrated J_SC_ (dashed lines) for (a) Control, (b) PBN, and (c) PBtA devices at different aging times (fresh, 2, 6, 24, and 165 h) under continuous 85 °C thermal stress in N₂ atmosphere.

**Figure S8** DLCP of ITO/ZnO/PM6:L8BO/MoOx/Ag and ITO/ZnO/Phenyl phosphonic acid (SAM treatment)/ PM6:L8BO/MoOx/Ag devices.

**Figure S9** OrbiSIMS mass spectra showing the characteristic ion fragments assigned to each material layer for depth profiling.

**Figure S10** Light intensity dependence of (a–c) the open-circuit voltage (V_OC_) and (d–f) short-circuit current density (J_SC_) for the (a, d) Control, (b, e) PBN, and (c, f) PBtA devices measured in the fresh state and after aging for 24 h and 165 h.

**Table S1** Ideality factor (n) and light intensity exponent (α) values for devices under different thermal aging time.

| Device type | Aging condition | *n* value | *α* value |
| --- | --- | --- | --- |
| Control | Fresh | 1.02 | 0.937 |
|  | Aged 24 h | 1.75 | 0.894 |
|  | Aged 165 h | 1.49 | 0.904 |
|  |  |  |  |
| PBN | Fresh | 1.07 | 0.911 |
|  | Aged 24 h | 1.01 | 0.903 |
|  | Aged 165 h | 1.08 | 0.911 |
|  |  |  |  |
| PBtA | Fresh | 1.05 | 0.901 |
|  | Aged 24 h | 1.65 | 0.898 |
|  | Aged 165 h | 1.50 | 0.907 |

**Figure S11** Photocurrent density (*J*_ph_) as a function of effective voltage (*V*_eff_) for the (a) Control, (b) PBN, and (c) PBtA devices measured in the fresh state and after aging for 24 h and 165 h. The charge dissociation probability (*P*_diss_) and charge collection probability (*P*_coll_) are extracted from the fittings at short-circuit and saturation regimes, respectively.

**Table S2** The exciton dissociation probability (*P_diss_*) and charge collection probability (*P_coll_*) values for devices under different thermal aging time.

| Device type | Aging condition | *P_diss_* (%) | *P_coll_* (%) |
| --- | --- | --- | --- |
| Control | Fresh | 96.1 | 87.0 |
|  | Aged 24 h | 85.2 | 67.8 |
|  | Aged 165 h | 94.7 | 79.4 |
|  |  |  |  |
| PBN | Fresh | 96.5 | 85.9 |
|  | Aged 24 h | 97.5 | 86.6 |
|  | Aged 165 h | 97.7 | 86.4 |
|  |  |  |  |
| PBtA | Fresh | 96.5 | 87.3 |
|  | Aged 24 h | 86.2 | 70.1 |
|  | Aged 165 h | 95.1 | 78.8 |

**Figure S12** Normalized transient photocurrent (TPC) decay dynamics for the (a) Control, (b) PBN, and (c) PBtA devices measured in the fresh state and after aging for 24 h and 165 h.

**Table S3** TPC decay parameters of the sample devices under different thermal aging time.

| Samples | Fitting parameters | | | | |
| --- | --- | --- | --- | --- | --- |
|  | τ_1_ (ns) | A_1_ | τ_2_ (ns) | A_2_ | τ_avg._ (ns) |
| Control-fresh | 112 | 0.988 | 2300 | 0.122 | 139 |
| Control-aged 24h | 200 | 0.989 | 2010 | 0.108 | 220 |
| Control-aged 165h | 175 | 0.988 | 2010 | 0.123 | 198 |
|  |  |  |  |  |  |
| PBN-fresh | 113 | 0.981 | 1100 | 0.192 | 132 |
| PBN-aged 24h | 113 | 0.932 | 550 | 0.684 | 143 |
| PBN-aged 165h | 113 | 0.932 | 540 | 0.684 | 142 |
|  |  |  |  |  |  |
| PBtA-fresh | 118 | 0.988 | 2000 | 0.122 | 141 |
| PBtA-aged 24h | 190 | 0.989 | 2010 | 0.108 | 210 |
| PBtA-aged 165h | 180 | 0.986 | 1900 | 0.138 | 204 |

**Figure S13** Simulated energy level diagrams of the BHJ active layer in (a–c) Control and (d–f) PBN devices in the fresh state (left panel), after 24 h aging (middle panel), and after 165 h aging (right panel) at 85 °C. The LUMO of the acceptor (blue lines) and HOMO of the donor (red lines) are shown as a function of position across the active layer. The ZnO cathode contact is at x = 0 and the MoO_x_ anode contact is at x = d (d = 100 nm). Energy is plotted relative to the acceptor LUMO at x = d. For Control devices, MoO_x_ impurity diffusion into the bulk (24 h) induces pronounced band-bending; subsequent migration toward the ZnO interface (165 h) creates intensified local band-bending that acts as an extraction barrier. In contrast, PBN devices effectively suppress bulk MoO_x_ diffusion, maintaining nearly flat energy levels throughout aging with only minor band-bending at the ZnO interface after prolonged aging, which strengthens the local electric field to aid carrier extraction.

**Table S4** Default parameters used in the device simulations.

| **Parameter** | **Value** |
| --- | --- |
| Temperature | 300 K |
| Relative permittivity of active layer | 3.5 |
| Thickness of the active layer | 100 nm |
| Electrical energy level gap | 1.31 eV |
| Effective density of states | 10^20^ cm^-3^ |
| Electron mobility | 10^-3^ cm^2^/Vs |
| Hole mobility | 10^-3^ cm^2^/Vs |
| Bimolecular recombination coefficient | 2.6×10^-12^ cm^3^/s |
| Electron capture coefficient | 2.6×10^-12^ cm^3^/s |
| Hole capture coefficient | 2.6×10^-12^ cm^3^/s |

**Table S5** The specific parameters that have been varied for the simulations of control and PBN devices at different aging times.

| **Parameter** | **Value** | | |
| --- | --- | --- | --- |
|  |  |  |  |
| **Control device** | **0 h** | **24 h** | **165 h** |
| Trap density in the bulk, *N*_t,bulk_ | - | 4.5×10^17^ cm^-3^ | 3.0×10^16^ cm^-3^ |
| Trap density near the cathode, *N*_t,int_ | - | 5.5×10^18^ cm^-3^ | 4.0×10^19^ cm^-3^ |
| Hole injection barrier at anode,  φan*𝜑an* | 0.0 eV | 0.07 eV | 0.10 eV |
| Electron injection barrier at cathode,  φcat*𝜑cat* | 0.13 eV | 0.0 eV | 0.13 eV |
| External series resistance, *R*_series_ | 0.4 Ωcm^2^ | 0.15 Ωcm^2^ | 0.9 Ωcm^2^ |
|  |  |  |  |
| **PBN device** | **0 h** | **24 h** | **165 h** |
| Trap density in the bulk, *N*_t,bulk_ | - | - | - |
| Trap density near the cathode, *N*_t,int_ | - | 2×10^18^ cm^-3^ | 3.5×10^18^ cm^-3^ |
| Hole injection barrier at anode,  φan*𝜑an* | 0.10 eV | 0.10 eV | 0.10 eV |
| Electron injection barrier at cathode,  φcat*𝜑cat* | 0.10 eV | 0.10 eV | 0.10 eV |
| External series resistance, *R*_series_ | 0.2 Ωcm^2^ | 0.2 Ωcm^2^ | 0.7 Ωcm^2^ |

**Figure S14** Photostability of Control, PBN-, and PBtA-treated devices under continuous 1-sun illumination in N_2_.

**Figure S15** PCE of the (a) PBN- and (b) PBtA-treated OSCs as a function of the concentration of passivation solutions.
